# Recent discoveries and advancements in research on **the Lyme disease spirochete *Borrelia burgdorferi*

**DOI:** 10.12688/f1000research.18379.1

**Published:** 2019-05-31

**Authors:** Christa Winslow, Jenifer Coburn

**Affiliations:** 1 Department of Microbiology and Immunology, Medical College of Wisconsin, 8701 Watertown Plank Road, Milwaukee, WI, 53226, USA; 2 Department of Medicine, Medical College of Wisconsin, 8701 Watertown Plank Road, Milwaukee, WI, 53226, USA

**Keywords:** Borrelia, B. burgdorferi, Lyme disease

## Abstract

This review highlights some of the highest-profile developments and advancements in the research on *Borrelia burgdorferi*, the Lyme disease spirochete, that have emerged in the last two years. Particular emphasis is placed on the controversy surrounding genus nomenclature, antigenic variation at the *vlsE* locus, genes involved in infectivity and virulence, membrane characteristics of *B. burgdorferi*, and developments in experimental approaches.

## Introduction 

*Borrelia burgdorferi* is an intriguing and unique bacterium. It is most notorious for being the primary causative agent of Lyme disease in North America ^1^. With more than 42,000 probable and confirmed cases reported to the Centers for Disease Control and Prevention (CDC) in 2017 and other estimates in the range of 300,000 cases annually in the US, Lyme disease is the most common arthropod-borne disease in North America and Europe ^2, 3^ (CDC, https://www.cdc.gov/lyme/datasurveillance/index.html). Case numbers have increased over the past several years and continue to be a growing concern as the range of the tick vector, *Ixodes scapularis* in particular, expands in North America ^4^ (CDC, https://www.cdc.gov/lyme/datasurveillance/index.html). Although antibiotic regimens are available and effective for treating the early stages of infection, Lyme disease often progresses without diagnosis ^5^. Serious long-term effects include arthritis, carditis, and neuroborreliosis ^5, 6^. Consequently, much research has focused on the development of a vaccine to administer to humans in endemic regions such as the Northeast and upper Midwest of the US ^7^.

*B. burgdorferi* is a motile, Gram-negative, pathogenic spirochete with a highly ** segmented genome composed of a linear chromosome and about 20 linear and circular genomic plasmids ^1, 8^. It exists in an enzootic life cycle alternating between specific *Ixodes* ticks and vertebrates ^9^. Humans can become incidental hosts through the bite of an infected tick ^9^. Whereas some *Borrelia* species, including *B. burgdorferi*, cause Lyme disease, other species cause relapsing fever, which is characterized by recurring episodes of fever and illness correlated with the bacterial burden in the bloodstream ^10^.

The focus of this article is to briefly highlight some of the major developments and advancements in understanding *B. burgdorferi* published within the last two years. As such, the content of this review is inherently selected on the basis of the authors’ interests and perspectives. Nevertheless, this article provides a summary of some of the most significant recent developments regarding genus nomenclature, antigenic variation at the *vls* locus, genes involved in infectivity and virulence, membrane chemistry, and advancements in laboratory techniques.

## Genus name and species 

A recent source of controversy and confusion in the spirochete world surrounds the genus name itself. Historically, *B. burgdorferi sensu lato* is a designation inclusive of all Lyme disease *Borrelia* species and includes *Borrelia afzelii* and *Borrelia garinii* species known to cause disease in Europe and Asia, whereas *B. burgdorferi sensu stricto* exclusively denotes *B. burgdorferi* ^9^. Lyme disease–causing and relapsing fever–causing spirochetes collectively resided in the single genus *Borrelia* since both clades have general microbiological features in common and genomic similarities (reviewed in 11). The clades do have differences regarding tick vector species, geographic distribution, ecology, transmission, and clinical manifestation and pathogenesis (reviewed in 11). In 2014, Adeolu and Gupta published a controversial proposition to divide the genus into *Borrelia* and *Borreliella*, the former including agents of relapsing fever and the latter denoting the Lyme disease–causing species, on the basis of average nucleotide identity (ANI) ^12^. This work aimed to identify conserved signature insertions/deletions and conserved signature proteins that could be used to distinguish between the groups. Their approach was criticized for presupposing differences between the clades in their analysis and overlooking the similarities between these groups, and the proposed naming system has been controversial and largely not adopted in the literature. In recent response to this work, Margos *et al*. outlined how ANI, though useful for determining relatedness between strains of a given species, is not appropriate for differentiating genera ^13^. Instead, they used pairwise analysis of percentage of conserved proteins (POCP) to determine whether there were differences significant enough to warrant the division of the *Borrelia* genus ^13^. The result was negative and rather suggested that *Borrelia* species exist more as a continuum ^13^. In an additional letter to the editor, Margos *et al*. itemized scientific criticisms and practical concerns about the genus split ^14^, which are largely and rather convincingly countered by Barbour *et al*. ^15^. A non-scientific yet compelling argument against the genus split is the confusion and public health concern associated with altering the name of a pathogen ^16^. Lending further backing to the reunification of the genus, a phyloproteomic analysis found insufficient functional differences between Lyme disease and relapsing fever clades to warrant individual genera ^17^. Annotated proteomes from species representing each of the five spirochete genera and 40,000 proteins representing key biological processes were compared by a series of methods to demonstrate more relatedness between Lyme disease–causing and relapsing fever–causing *Borrelia* than exists between species of other genera ^17^. Of additional note, the genome of *Borrelia anserina*, an avian-associated species, was recently fully sequenced ^18^ and new species continue to be discovered, such as *Borrelia mayonii*, which was isolated in the North Central US in 2016 ^19, 20^ and echidna-associated *Candidatus Borrelia tachyglossi* ^13, 21^. It is our opinion that the *Borrelia* genus should not be split into *Borrelia* and *Borreliella*, because if different scientific approaches do not yield the same results, no firm conclusion can be drawn at this time. Perhaps as more species are discovered and characterized, the scientific merit or detriment of splitting the genus will become clearer.

## Antigenic variation of VlsE 

*B. burgdorferi* is able to establish persistent infection in immunocompetent hosts (as reviewed in 6, 9). Antigenic variation in the *vlsE* locus is thought to play a large part in immune evasion since VlsE is expressed during mammalian infection but not within the tick vector ^22^, and *Borrelia* strains lacking *vlsE* are unable to maintain infection in immunocompetent mice as opposed to severe combined immune deficiency (SCID) mice ^23– 25^. The *vls* system, encoded on lp28-1 (in B31-type strains), includes a series of silent cassettes upstream of the *vlsE* promoter ^26, 27^. Through a segmental gene conversion mechanism, unidirectional transfers of sequences from the silent cassettes to the *vlsE* variable regions generate chimeric antigens during infection ^28^. Research on the *vlsE* locus-switching mechanism has been impaired by the necessity for mammalian infection, coupled with the limitations of sequencing strategies that align short reads, and therefore cannot capture the nuances in genomic changes during infection ^26, 29^. In a groundbreaking study, Verhey *et al*. advanced the field by applying PacBio long-read sequencing to sequence the *vls* locus ^26^. These long reads are sensitive to single-nucleotide polymorphisms and reduce the ambiguity associated with contig assembly ^26^. These authors also built Variable Antigen Sequence Tracer (VAST), a powerful program that analyzes the data and aligns the *vls* locus to the reference sequence. This technique was applied to study *vlsE* switching in the context of infection by injecting wild-type (WT) and SCID mice with *B. burgdorferi*, harvesting multiple tissues at various time points, and sequencing the re-isolated spirochetes ^26^. A number of interesting findings resulted and build off previous findings ^30^. First, the base-change frequencies of *vlsE* were dramatically higher in the WT mice in comparison to SCID mice, confirming earlier work suggesting that pressure from the acquired immune response facilitates detection of *vlsE* variants ^23– 25^. Second, they observed that 99.6% of *vlsE* variants were found to be unique in each tissue in any given mouse ^26^. This sheds light on the dissemination process in mice. During infection, *B. burgdorferi* replicates and disseminates through the bloodstream to different tissues ^31^. The uniqueness of the *vlsE* variants in each tissue suggests that *B. burgdorferi* cells colonize a tissue, and reside there, rather than re-entering the bloodstream and colonizing additional tissues ^26^. Third, lack of obvious preference toward switching at the 5′ or 3′ end of the gene suggests that *vlsE* switching occurs stochastically rather than in a biased fashion ^26^. Fourth, three-dimensional structure mapping of the VlsE amino acid changes found in WT mice versus SCID mice showed that infection in WT mice required switching on surface-exposed loops as opposed to switching in the hydrophobic α-helices ^26^. These hydrophobic α-helices are putatively involved in dimerization, suggesting that functional dimerization of VlsE is necessary *in vivo* and therefore conserved ^26^. This work was performed with *B. burgdorferi* strain B31, and similar results were obtained with strain JD1, which differs in several respects: it lacks 17 base-pair direct repeats in the *vls* system, its silent cassettes are arranged differently, the inverted repeat sequences at the *vls* locus are different, and the *vls* sequences themselves are different ^32^. Investigations into other features of the *vls* locus, such as the long inverted repeat that resides in the *vlsE* promoter, have been undertaken through the construction of mini- *vls* plasmids ^33^. This work suggests that *vlsE* switching occurs in *cis* and that the inverted repeat is not required for switching to occur ^33^. The mechanistic details whereby *vls* switching occurs and how VlsE protects *B. burgdorferi* from the host antibody response remain of great interest for further investigation.

## Infectivity and virulence attributes distinct from antigenic variation 

The genome of *B. burgdorferi* is relatively small and does not encode enzymes key to several metabolic processes ^8^. This leaves *B. burgdorferi* dependent on its host for many nutrients and presumably renders essential many of the genes it does possess. Genes essential for survival in the tick vector or in the mammalian host (or both) continue to be identified. One such gene is *bb0210*, which encodes Lmp1, an antigenic outer-membrane protein ^34^. Lmp1 appears to be a multifunctional protein (similar to BBK32 ^35– 37^) with roles that include adhesion at the middle domain ^38, 39^, immune evasion for persistent infection ^40^, and tissue tropism during infection ^41^. Although three domains of Lmp1 were previously recognized ^41^, the necessity of the individual domains for a mammalian infection has not been fully investigated. In a recent publication, Zhuang *et al*. ^34^ provide evidence for reduced bacterial burden of the *Δlmp1* mutant in mouse tissues at 3 weeks post-infection, the requirement for both the N-terminal and middle domains in tick-to-mouse transmission, the existence of multiple Lmp1 species, and the degradation of Lmp1 by the periplasmic serine protease BbHtrA ^34^. Further investigation of the complexities of the *in vivo* roles of the Lmp1 protein and its fragments is warranted.

In a separate inquiry, recent work demonstrated that BB0405 is necessary for mouse infection ^42^. This is interesting because BB0405 and BB0406 may be pore-forming proteins ^43^ that are immunogenic, highly paralogous (differing by a five–amino acid deletion in the N-terminal region of BB0405 and a nine–amino acid deletion in the C-terminal region of BB0406), and co-transcribed ^8, 42^. A null mutant of *bb0405* effectively disrupted production of both BB0405 and BB0406 at the protein level. The *bb0405* mutant was complemented with either *bb0405* or *bb0406* by insertion into a different replicon, circular plasmid 26 (cp26). When these strains were used to infect mice, culture-positive tissue samples were recovered for the *bb0405*-complemented, but not for the *bb0405* mutant or *bb0406*-complemented, strains. This confirms that BB0405 is essential in mammalian infection but the role of BB0406 remains unknown. Despite their similarities, BB0405 and BB0406 do not appear to be co-expressed, as evidenced by reactivity with baboon sera collected at different times post-infection. The mechanism underlying this apparent co-transcription without co-expression of these genes warrants further investigation of the transcriptional and translational regulators critical to the infection process.

## Membrane characteristics 

The membrane composition and characteristics of *B. burgdorferi* differ markedly from those of other Gram-negative bacteria. For example, the outer and inner membranes are separated by periplasm and peptidoglycan, as is usual, but also by periplasmic flagella ^44^. *Borrelia* species ** lack lipopolysaccharide, and *B. burgdorferi* has an unusually large number of lipoproteins on its outer membrane ^45, 46^. Recent work has investigated the existence of lipid rafts in both the outer and inner membranes. Although this work was performed on outer- and inner-membrane fractions prepared *in vitro*, data from liquid chromatography, anisotropy and fluorescence resonance energy transfer (FRET) analysis, mass spectrometry, and proton nuclear magnetic resonance ( ^1^H-NMR) experiments have led to some interesting conclusions ^47^. Lipid rafts have previously been demonstrated in the outer membrane, but there is now evidence for lipid rafts in the inner membrane ^48^. The inner membrane has a different quantity of cholesterol glycolipids and more unsaturated bonds in the acyl chains of the phospholipids than the outer membrane, causing it to have a lower propensity to lipid raft formation than the outer membrane ^47^. As expected, the proteomic profiles of the membranes differ such that the inner membrane harbors more transport proteins and signaling proteins whereas the outer membrane harbors porins and lipoproteins, at least some of which interact with the host ^47^. In another ambitious study, the localization of the entire lipoproteome of *B*. *burgdorferi* was determined ^49^. This was accomplished by using an algorithm to identify putative lipoproteins in the *B. burgdorferi* genome, then generating a 125-member plasmid library encoding C-terminally His-tagged lipoproteins, and transforming these constructs into *B. burgdorferi* for analysis ^49^. Transformants were screened for tagged lipoprotein localization by proteinase K digestion, membrane fractionation, pronase digestion, and multidimensional protein identification technology (MudPIT) mass spectrometry ^49^. Although there are always caveats to the use of overexpressed tagged proteins, some of the results are still striking. Of the 125 lipoproteins examined, 86 were surface-exposed, 31 were in the inner membrane and exposed to the periplasm, and only eight were in the outer membrane and exposed to the periplasm ^49^. This is in contrast to other Gram-negative bacteria, which have higher proportions of lipoproteins in the outer membrane exposed to the periplasm, again highlighting the uniqueness of *B. burgdorferi* ^49^. Although the significance of differential lipoprotein expression and localization during tick and mammalian infection is one of the most established tenets in *Borrelia* biology, a comprehensive study had never been performed. Understanding the membranes of *B. burgdorferi* and their components may well lead to identification of novel vaccine targets in the future.

## Progress in the development of experimental approaches 

Genetic manipulation of *B. burgdorferi* has been greatly hindered by a number of factors, including slow growth, loss of genomic plasmids during *in vitro* cultivation, lack of a minimal defined medium, a highly A/T-rich genome, and lack of homology in many cases to the genes and proteins of model organisms ^8, 50, 51^. Therefore, many of the tools and techniques used for the manipulation of bacteria such as *Escherichia coli* have not been tractable in *B. burgdorferi*. However, great strides have recently been made to expand the toolbox. In 2016, the first report of the application of transposon sequencing (Tn-seq) in the context of *B. burgdorferi* infection was published ^52^. This study used the signature-tagged Mariner *Himar1* transposon library in an infectious strain background to address the utilization of various carbohydrate sources *in vitro* and to identify genes important during mammalian infection ^52– 55^. The following year, Tn-seq was used to identify genes involved in resistance to nitric oxide, hydrogen peroxide, and *tert*-butyl hydroperoxide *in vitro* ^56^. These studies pave the way for future high-throughput screens both *in vitro* and *in vivo* for *B. burgdorferi*.

An additional advance in *B. burgdorferi* research is in the introduction of constitutive promoters of different strengths. Although strong promoters from the *flaB* and *flgB* genes have been used historically, transcriptome data have now been used to identify constitutively weak (P _0526_) and moderate (P _0826_, P _resT_, P _0031_, and P _0026_) promoters which will be useful for expressing more biologically relevant levels of a gene of interest ^57^. Furthermore, monomeric fluorescent markers have been adapted for *B. burgdorferi*, including cyan, green, yellow, red, and infrared fluorescent proteins. The infrared marker requires the addition of exogenous biliverdin, but the excitation wavelength is less toxic to cells than other markers and results in less autofluorescence. These markers will inevitably prove useful in co-localization studies and other research and allow simultaneous imaging of up to four distinct proteins in a cell ^57^. In the same publication, the options for antibiotic markers were expanded. Historically, kanamycin ( *aphI*), gentamicin ( *aacC1*), and streptomycin ( *aadA*) resistance genes have been the available markers of choice ^57^. Now resistance markers that enzymatically inactivate the translation inhibitors hygromycin B and blasticidin S have been adapted for use in *B. burgdorferi* ^57^. Since these drugs are not used clinically and exhibit minimal cross-resistance with the other markers, they can be exploited for the genetic manipulation of *B. burgdorferi* ^57^. These laboratory tools will facilitate further research into *B. burgdorferi* infection and physiology, which will likely contribute to novel strategies to prevent and treat Lyme disease.

## Conclusions

Although the word limit of this review requires that we not include the vast majority of recent publications, it is hoped that some major themes, advancements, and future directions are clear. Controversy surrounding genus nomenclature persists for now but hopefully will be resolved so energy can again be directed toward the biology of the organisms and the diseases they cause. Further research into the antigenic variation system encoded at the *vls* system is now enabled by PacBio long-read sequencing and the VAST program to track genetic changes in *B. burgdorferi*. The mechanisms behind this unique segmental gene conversion system, and therefore the antigenic variation system that allows *B. burgdorferi* to maintain infections, deserve more study. We look forward to seeing how this area of research unfolds and its implications for the biology of the Lyme disease agents. Similarly, we anticipate the identification and investigation of more genes essential to *B. burgdorferi* infectivity and virulence. In addition to increasing our understanding of *B. burgdorferi* biology, these studies may direct investigation of future vaccine targets. We also sought to highlight the protein and lipid characteristics of the *B. burgdorferi* membranes. In light of its existence as an extracellular pathogen, these characteristics and dynamic changes are significant for interactions of *B. burgdorferi* with its environment, be it tick or vertebrate. By extension, general understanding of the physiology of *B. burgdorferi*, obtained through the applications of newly pioneered laboratory techniques, will uncover the secrets of this fascinating spirochete.

## Abbreviations 

ANI, average nucleotide identity; SCID, severe combined immune deficiency; Tn-seq, transposon sequencing; VAST, Variable Antigen Sequence Tracer; WT, wild-type
